# A passion for paediatrics and infection control

**DOI:** 10.4102/sajid.v37i2.380

**Published:** 2022-02-14

**Authors:** Angela Dramowski

**Affiliations:** 1 Department of Paediatrics and Child Health, Faculty of Medicine and Health Sciences, Stellenbosch University, Cape Town, South Africa

As an infectious disease paediatrician and infection preventionist, my research is inspired by the challenges and disease burden I encounter in clinical practice. Antibiotic-resistant infections are increasingly important causes of morbidity and mortality in newborns and children in low- and middle-income countries. My research focuses on the epidemiology, diagnosis, treatment and prevention of neonatal and paediatric infections in South Africa (SA) and Africa. I am passionate about patient safety, infection prevention and data-driven quality improvement in the care of hospitalised neonates and children. Since 2008, I have been based at the Department of Paediatrics and Child Health at Stellenbosch University and Tygerberg Hospital.

Paediatric infectious diseases (PID) has been my interest and passion from the early days of my medical career as an undergraduate student at the University of Cape Town (UCT). My love for infectious diseases (ID) and paediatrics was sparked by several inspiring clinicians, including Arderne Forder, Gary Maartens, Cas Motala and Gregory Hussey, among others. I had the privilege of doing a ‘deep rural’ internship at Shongwe Hospital on the border of Mpumalanga and Swaziland, which provided a unique ID experience with patients suffering from malaria, bilharzia, rabies, tetanus and snake bites, and cemented my determination to subspecialise in ID. Following a brief stint back in Cape Town for community service at Red Cross Children’s Hospital and Retreat Day Hospital (plus a few weeks off to recover from occupational tuberculosis [TB]), I left SA to spend a year in the United Kingdom working in neonatology and paediatrics.

Suitably convinced that paediatrics in resource-limited settings was my calling, I headed back to SA for four hectic years of paediatric registrar training at WITS/Chris Hani Baragwanath Hospital. Sithembiso Velaphi’s passionate teaching on neonatal sepsis and a research opportunity in paediatric HIV research with Tammy Meyers, Ameena Goga and Ashraf Coovadia stimulated my interest in clinical research. In 2008, I was awarded a one-year Fogarty-National Institutes of Health (NIH) International Clinical Research Fellowship to work with Professors Mark Cotton, Helena Rabie and Simon Schaaf at the Family Centre for Research with Ubuntu (FAMCRU). Three years later, I became the first PID fellow to graduate from Tygerberg Hospital/Stellenbosch University.

During my fellowship training, I realised the need for intensified research in Infection Prevention and Control (IPC) in Africa. After graduation, I joined the Stellenbosch University’s Academic Unit for IPC, one of the only specialised IPC units in the country, working under the mentorship of Shaheen Mehtar. A key output of my time at the UIPC was publishing their book: *IPC: A guide for healthcare workers in low-resource settings.* Having identified a gap in clinical research in IPC, I embarked on a Doctor of Philosophy (PhD) on healthcare-associated infection (HAI) in hospitalised children in Cape Town, with Mark Cotton and Andrew Whitelaw as supervisors. I also benefitted from an opportunity to engage in IPC internationally, as the first South African selected for the Society for Healthcare Epidemiology of America’s International Ambassadors programme. Through this programme, I met and befriended Susan Coffin (PID clinician-researcher from the Children’s Hospital of Philadelphia). Thanks to the fantastic support and mentorship from Mark Cotton, Susan Coffin and many other ‘giants of ID/IPC’, I was awarded a career development grant in 2018 from the NIH’s Emerging Global Leader programme. The grant allowed me to research the prevention of neonatal sepsis in low-resource settings. This award has been truly transformative in that it afforded me an opportunity to conduct full-time research with several ‘thought-leaders’ in the field of PID.

As my research expertise grew and grant-writing ability developed, my funding success rate improved, and my research network expanded considerably, allowing me to work as principal investigator on 12 local and international studies to date, including two clinical trials. As my own research career advanced, I have been privileged to mentor and supervise 30 junior colleagues’ research projects to date and increasingly collaborate across several disciplines, including neonatology, microbiology, virology, pharmacology, human nutrition, nursing and data science. A key partnership with the Federation of Infectious Disease Societies of Southern Africa (FIDSSA) and the National Institute for Communicable Diseases saw the launch of the national neonatal sepsis task team in 2019 to strengthen surveillance, diagnosis, treatment, and prevention of neonatal sepsis in SA. My career has also been enriched by collaborations with a variety of local, national and international academic institutions, non-profit organisations (the Global Antibiotic Research and Development Partnership [GARDP], Bettercare, the Infection Control Africa Network [ICAN]) and advocacy groups (TB Proof).

In the 10 years since qualifying as a paediatric ID sub-specialist, my career as a clinician-researcher has grown through the unwavering support of senior colleagues and mentors. My advice to colleagues for a fulfilling career in ID is to be inquisitive, identify a research or clinical practice ‘gap’ to develop, find an experienced, enthusiastic mentor and actively ‘pay it forward’ by supporting and mentoring their junior colleagues.

‘There are two primary choices in life: to accept conditions as they exist, or accept the responsibility for changing them.’ – Denis Waitley

